# Clinical decision support system impact on diagnostic imaging appropriateness and value-driven outcomes in a Singapore emergency department: Protocol of a pragmatic controlled before-after study

**DOI:** 10.1371/journal.pone.0336801

**Published:** 2025-12-26

**Authors:** Yi Xiang Tay, Shane J. Foley, Ronan Killeen, Jeremy C.P. Wee, Ivan S.Y. Chua, Marcus E.H. Ong, Peter Doran, Robert Chun Chen, Lai Peng Chan, May San Mak, Jonathan P. McNulty

**Affiliations:** 1 Radiography and Diagnostic Imaging, School of Medicine, University College Dublin, Belfield, Dublin, Ireland; 2 Radiography Department, Allied Health Division, Singapore General Hospital, Singapore; 3 St Vincent’s University Hospital, Dublin, Ireland; 4 School of Medicine, University College Dublin, Belfield, Dublin, Ireland; 5 Department of Emergency Medicine, Division of Medicine, Singapore General Hospital, Singapore; 6 Duke-NUS Graduate Medical School, Singapore; 7 Clinical Research Centre, School of Medicine, University College Dublin, Belfield, Dublin, Ireland; 8 Department of Neuroradiology, Division of Radiological Sciences, Singapore General Hospital, Singapore; 9 National Neuroscience Institute, 1 Hospital Boulevard, Singapore; 10 Department of Musculoskeletal Radiology, Division of Radiological Sciences, Singapore General Hospital, Singapore; Tehran University of Medical Sciences, IRAN, ISLAMIC REPUBLIC OF

## Abstract

Modern medicine relies heavily on diagnostic imaging because of its beneficial role in the healthcare chain. Imaging rates are increasing globally and are expected to continue to rise in the future. In tandem with this phenomenon is the increase in inappropriate imaging that adversely affects the provision of healthcare and increases the risks to patients. To date, effective interventions to reduce inappropriate imaging have shown conflicting results due to various underlying implementation designs and strategies. The aim of this controlled before-after study is to assess the impact of a clinical decision support system (CDSS) on appropriateness rates of X-ray cervical and lumbar spine in the emergency department (ED) compared to the existing practice without one, while also exploring associated outcomes. In this study, a CDSS will be carried out using implementation science principles to enhance evidence uptake with institutional medical board-approved imaging referral guidelines embedded. Recruited ED physicians will be allocated into control and intervention groups. The control group will continue the routine practice of “do nothing” for four months, while the intervention group will have a two-month CDSS intervention after an initial two-month routine practice of “do nothing”. The difference between the baseline and post-implementation appropriateness rate of imaging will be compared. This study will also evaluate the impact of CDSS on clinical effectiveness, cost avoidance, radiation doses, and sustainability. Findings from this before-after study will provide a rigorous, pragmatic test of the impact and effect of radiological CDSS as an evidence-based intervention to reduce inappropriate imaging in the ED.

## Introduction

Diagnostic imaging has seen significant change over time. Since Wilhelm Conrad Roentgen’s discovery of X-rays, the field has evolved with the introduction of sophisticated equipment and new diagnostic imaging techniques, with many more emerging technologies, such as artificial intelligence, that will continue to change the way we acquire and process images [1]. Modern medicine relies heavily on diagnostic imaging, with clinicians perceiving it as a high-value tool with both clinicians and patients benefiting from the diagnostic insights provided by imaging procedures [2,3]. Diagnostic imaging embedded within the healthcare system plays a multitude of roles in the healthcare chain, such as prevention, detection, and diagnosis of diseases; delivery and monitoring of therapy; prognosis; and many others [4]. Indeed, with such a multitude of roles, the use of diagnostic imaging is increasing globally and is expected to rise more in the future [2,5,6].

However, heavy reliance on diagnostic imaging raises serious concerns. Economically, diagnostic imaging is expensive especially for advanced imaging techniques such as computed tomography (CT) and magnetic resonance imaging (MRI) [7]. In light of escalating healthcare expenditures and limited resources, the continued growth and demand of diagnostic imaging is unsustainable. Furthermore, there are potential negative ramifications for patients, such as the detection of benign incidental findings requiring further unnecessary workup, resulting in unwarranted patient anxiety from the aforementioned processes [8]. More importantly, some diagnostic imaging techniques, such as X-rays and CT, involve ionising radiation, which subjects patients to additional radiation exposure, making radiation protection a crucial aspect of the practice.

There are an abundance of studies that highlight the effects of ionising radiation on the risk of cancer and mortality. A *BMJ* study found a linear increase in the relative cancer risk with increased radiation exposure, along with evidence of a steeper dose-response relationship at lower doses compared to the full dosage spectrum [9]. A separate population-based cohort study involving over 12 million South Korean youths revealed that the overall cancer incidence was higher in individuals exposed to diagnostic low-dose ionising radiation compared to those who were not exposed [10]. Similar findings were also demonstrated in other studies [11,12]. Notably, a recent study revealed that patients receiving relatively higher doses of radiation had a higher mortality rate within the first two years, with nearly one-third to half still alive a decade after exposure, potentially facing the effects of radiation [13]. These studies underscore the necessity of radiation protection for patients. Alongside these issues, low-value and/or inappropriate imaging exposes patients to a carcinogenic cancer risk that may manifest decades after exposure, a possibility that clinicians often overlook due to its seemingly distant nature [14].

Low-value and/or inappropriate imaging is prevalent, with a scoping review indicating that the percentage of low-value imaging ranged from 2% to 100% for inappropriate or unnecessary procedures [15]. Many factors, such as organisation culture, communication, competence, expectations, defensive medicine, roles and responsibilities, referral quality, and time constraints, exacerbate the problem of low-value imaging [16]. A systematic review of the cost associated with low-value imaging determined that the cumulative costs for such procedures totalled billions of dollars worldwide annually [17]. This warranted immediate attention to mitigate the growth of low-value and/or inappropriate imaging.

The emergency department (ED) is an area that has demonstrated a trend in increasing imaging usage [18–20]. Furthermore, there is growing evidence that low-value and/or inappropriate imaging is widespread in the ED. A recent study on the appropriateness of ultrasound, CT, and MRI in the ED of an urban academic centre revealed that the proportion of inappropriate imaging referrals ranged from 29.1% to 59.4%, depending on the imaging modality [21]. Similarly, in an early retrospective study in an ED in Singapore, it was found that 36.2% of the patients had inappropriate lumbar spine X-rays performed [22]. Furthermore, a 3-year study period’s data analysis revealed that every 18 eligible encounters included a low-value imaging study [23]. Simultaneously, ED physicians also reported imaging overuse in the ED as a problem, which is driven by several factors (i.e., defensive medicine, elderly patients) as illustrated in a systematic review by Tung et al [24,25].

Accordingly, various efforts have been explored to optimise diagnostic imaging in the ED [26]. Imaging referral guidelines have been identified as a tool that can facilitate optimisation of diagnostic imaging usage in the ED [26,27]. Imaging referral guidelines include those published by radiology societies like the American College of Radiology (ACR) Appropriateness Criteria (AC), the European Society of Radiology (ESR) iGuide, and the Royal College of Radiologists (RCR) iRefer, as well as those from non-radiological societies like Choosing Wisely (CW). A recent systematic review on the impact and effect of imaging referral guidelines highlighted that these guidelines increase the value of radiology in the healthcare system and improve the quality of healthcare and outcomes while reducing healthcare costs [28]. To further increase the impact and effect of these guidelines, they can be integrated with clinical decision support systems (CDSS) to facilitate point-of-care advice and feedback on imaging referral behaviour [29]. According to a recent study, the implementation of CDSS may lead to a reduction in multimodality imaging referrals and advanced imaging volume, while also reducing the effective dose per patient and carbon emissions [30]. Additionally, a systematic review on the effectiveness of CDSS for imaging of central nervous system injuries also showed a decrease in the use of CT and an increase in physicians’ adherence to guidelines [31].

Despite the positive utility of these findings from multiple studies, there is currently no requirement for physicians to consult imaging referral guidelines/CDSS prior to making imaging referrals in the Asian context. While the Protecting Access to Medicare Act in the United States mandates CDSS consultation for all advanced imaging, uptake of this evidence-based intervention (EBI) is still lacking, especially in Asia [32]. This was reflected in the systematic review by Tay et al., where the majority of the studies included were from the United States and Europe [28]. Various editorials from across Asia, including Korea, Singapore, and Taiwan, have advocated for strategies to resolve the growing problem of radiology manpower shortage, whereby one proposed solution is to reduce unnecessary imaging, which not only burdens radiologists but also the aforementioned problems [33–35].

However, barriers such as cost, stakeholders buy-in, lack of use, awareness, access, familiarity, usefulness, and motivation have hindered the uptake of EBIs like CDSS [36]. Implementation science can help address barriers and facilitate uptake of EBIs [37]. Despite the existence of numerous studies exploring the use of CDSS in clinical settings, this pragmatic controlled before-after study integrates an EBI (CDSS) developed using implementation science principles to enhance evidence uptake. The overall aim is to assess if the introduction of a CDSS will impact the appropriateness of X-ray cervical and lumbar spines in the ED compared to the existing practice without one. We will also evaluate the associated outcomes following the CDSS implementation.

1. Adherence to evidence-based imaging guidelines
2. Clinical effectiveness
3. Cost avoidance
4. Radiation dose
5. Sustainability

Additionally, we will use a controlled before-and-after study design to help attribute observed effects to the CDSS intervention with reasonably confident conclusions.

## Methods

### Design and setting

This implementation research is designed to be a single-centre, pragmatic, controlled before-after study. The study received ethics waivers from the SingHealth centralised institutional review board (CIRB) (CIRB Ref: 2020/2941). Consent was waived by the ethics committee.

The study will take place in the largest tertiary hospital in Singapore, which receives an ED attendance rate of approximately 120,000 annually. The ED provides comprehensive 24-hour emergency medicine (EM) services for a wide range of emergencies, including major trauma. It offers direct access to 24/7 laboratory and diagnostic imaging services. The ED care team is comprised of EM specialists (associate consultants and above) and non-EM specialists (residents, medical officers, resident physicians, etc.).

### Sample size

Based on previous data [22,38], we assume an estimated 20% of exams are inappropriate across all imaging procedures (based on previous studies conducted locally). In this study, we seek to demonstrate a reduction in inappropriate exams using a before and after design. Data from independent before and after groups will be compared using t-test. To achieve a power of 0.9, with an alpha error of 0.05 and an effect size of 0.3, we will need 468, therefore, 235 per group, before and after the intervention.

The primary analysis is conducted at the physician (cluster) level. For each physician, we compute the mean appropriateness rate in the pre-implementation and post-implementation phases and analyse the within-physician change. Power calculations were therefore based on a paired t-test of physician-level means, which is asymptotically equivalent to tests for differences in proportions when binary outcomes are summarised at the cluster level.

Sources of previous studies conducted locally:

1) Tay YX, Chan LL, Than SR, et al. Appropriateness of lumbar spine radiography and factors influencing imaging ordering patterns: paving the path toward value-driven health care. Int J Qual Health Care. 2023;35(2):mzad021. doi:10.1093/intqhc/mzad021
2) Tay YX, Foley SJ, Killeen R, et al. Positivity rates and subsequent patient dispositions after utilisation of cervical spine imaging referral guidelines in Singapore. Insights Imaging. 2025;16(1):170. Published 2025 Aug 8. doi:10.1186/s13244-025-02048-9
3) Tay YX, Foley SJ, Ong ME, et al. Using evidence-based imaging referral guidelines to facilitate appropriate imaging: Are they all the same?. Eur J Radiol. 2025;183:111933. doi:10.1016/j.ejrad.2025.111933

### Characteristics of participants

A purposive sample of junior ED physicians will be approached via the ED Trauma lead. Inclusion criteria will include non-specialist EM physicians, and exclusion criteria will include non-specialist ED physicians who are unable to commit to the study period of 4 months (i.e., rotating/posting out of the ED within the study timeframe). Only non-specialists ED physicians will be recruited as they are the primary care providers for most of the ED patients and also account for the majority of the X-ray imaging referrals from the ED. Additionally, they are mostly assigned for clinical duty in the P3 and P4 sections of the ED (patient acuity category status P3: ambulant patients with minor emergencies and P4: non-emergency cases), where a substantial volume of X-ray cervical and lumbar spine are requested [38]. Notably, a previously conducted study in the same ED revealed that non-specialists accounted for a greater proportion of inappropriate orders as compared to specialists [22]. Consequently, this navigated us from the pitfall of recruiting physicians with a low baseline percentage of inappropriate imaging requests, which limits the potential for reduction [39].

This study will not employ randomisation or blinding due to its pragmatic design. The research team will take informed consent verbally prior to the study’s commencement, following an introduction and briefing.

### Comparator and intervention

**Fig 1:**
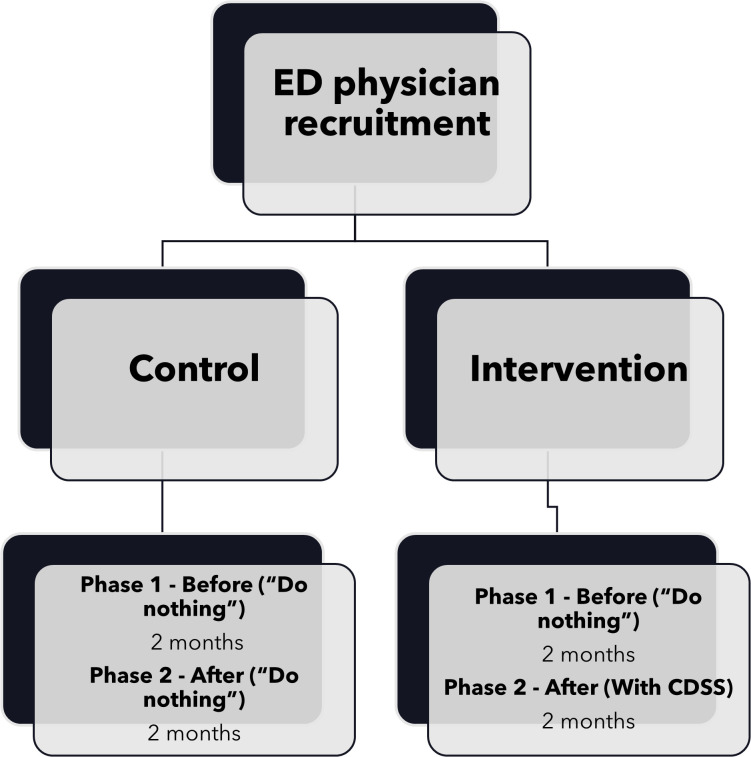
Flow chart and timeline of the study.

Recruited ED physicians will be allocated into the control and intervention groups based on their starting posting date in the ED (e.g., 2^nd^ quarter, 3^rd^ quarter etc). Fig 1 illustrates the flowchart and timeline for the respective groups involved in the study.

In the first phase for both groups, ED physicians will follow the usual clinical practice (“Do nothing”) of making imaging referrals without consulting CDSS. Both groups will be informed during their onboarding on the availability of imaging referral guidelines in the training handbook and also on the intranet. Specifically, the physicians in the control group will not receive any CDSS introduction/orientation and no additional imaging referral guidelines education beyond the standard onboarding process. Simultaneously, a retrospective analysis of the electronic medical records will be performed.

The study included consecutive patients referred to the ED’s radiology department for cervical and lumbar spine radiography by the cohort of recruited ED physicians. Patients who had imaging referrals from general practitioners will be omitted due to our lack of access to the clinical notes for this particular cohort. For each case, the initial clinical diagnosis (reasons for the referral), the ordered procedure, patient demographics (age and gender), and ED clinical notes will be collected. To enhance the accuracy of analysing each imaging referral, the initial clinical diagnosis will be integrated with the corresponding ED clinical notes, including signs, symptoms, clinical examination, and red flags. This integration will result in a more comprehensive “indication(s)” for the imaging procedure. The appropriateness of each imaging referral will be determined with reference to localised and institutional approved imaging referral guidelines built on imaging referral guidelines published by ACR, ESR and RCR (Refer to Table 1). The records will be archived to REDCap (Research Electronic Data Capture) hosted by SingHealth for subsequent analysis.

**Table 1:** Imaging referral guidelines* for X-ray Cervical and Lumbar Spine.

Procedure	Clinical condition	Appropriate Imaging Criteria
X-ray Cervical Spine	Acute trauma	1. Patient age is > 65 years old 2. Patient GCS score <15 3. Patient is intoxicated 4. Patient has midline spinal tenderness 5. Presence of focal neurological deficit 6. Presence of paraesthesia in extremities 7. Patient is unable to actively rotate neck left and right to 45 degrees 8. Not possible to test range of neck movement during patient assessment 9. Presence of dangerous mechanism of injury – fall from >1 meter/5 stairs 10. Presence of dangerous mechanism of injury – axial load injury 11. Presence of dangerous mechanism of injury – high speed motor vehicle accident 12. Presence of dangerous mechanism of injury – rollover motor vehicle accident 13. Presence of dangerous mechanism of injury – motor vehicle accident with ejection 14. Presence of dangerous mechanism of injury – motorised recreational vehicles 15. Presence of dangerous mechanism of injury – bicycle collisions
	Non-traumatic neck pain	1. Patient has history and/or current malignancy 2. Patient has prior cervical spine surgery 3. Presence of symptoms for spinal cord injury 4. Patient is suspected to have an infection 5. Patient has history of IV drug use 6. Patient has systemic disease including spondyloarthritis and diffuse idiopathic skeletal hyperostosis 7. Presence of intractable pain despite therapy 8. Patient has tenderness to palpation over a vertebral body 9. Presence of neurological deficits 10. Presence of radiculopathy 11. Presence of neck pain
X-ray Lumbar Spine	Low back pain (LBP)	1. Patient age is > 65 years old 2. Patient is osteoporotic 3. Patient has chronic steroid use 4. Patient has low velocity trauma 5. Patient is suspected to have metastatic disease 6. Patient is suspected to have an infection 7. Patient is immunosuppressed 8. Patient is a surgical candidate with persistent or progressive symptoms during or following 6 weeks of conservative management 9. Patient has prior lumbar surgery with new or progressive symptoms or clinical findings 10. Patient has trauma of dangerous mechanism

*Approved by Institution’s Medical Board.

During the second phase of the study, the ED physicians in the control group will continue their usual clinical practice of ordering imaging without consulting CDSS (“Do nothing”). We will study the referral practices of the ED physicians in the control group over a consecutive period of two months, using a pragmatic approach. We will perform a retrospective analysis of the electronic medical records, following the same approach as in Phase 1.

Conversely, the ED physicians in the intervention group will receive individual access to a CDSS after a two-month period (Phase 1 – No CDSS intervention), and they will continue clinical practice using the standalone CDSS, which will incorporate localised and approved institutional imaging referral guidelines. The CDSS adopted in this study will be xWave CDS (xWave Technologies Ltd., Dublin, Ireland), which is a cloud-based Software as a Service (SaaS) platform with ISO27001 certification and developed in partnership with both the ESR iGuide and RCR iRefer. xWave CDS is fully hosted in Amazon Web Services (AWS) (Seattle, USA), and access is through the public internet. There is no hardware required onsite other than devices to access a web browser. Approval will be sought from the local hospital information technology team to whitelist the xWave CDS uniform resource locator (URL) for use by physicians on the hospital computer system. As the CDSS is not integrated into the Computerised Provider Order Entry (CPOE) ordering process, physicians will be required to toggle between the CPOE platform and xWave CDS. Modifications will be made to the xWave CDS user interface to incorporate elements that were deemed user-friendly as highlighted by local ED physicians during a previous focus group study [40]. This includes consolidating imaging referral guidelines onto a single page and replacing drop-down boxes with checked boxes.

Briefing slides and an introduction/orientation to the CDSS will be provided to ED physicians utilising the CDSS prior to the start of this second phase (Phase 2). The briefing slides will be curated by the vendor project manager and the principal investigator (PI) and subsequently be reviewed by the senior ED physicians for comprehensibility to ensure that the materials are well-defined and well-understood. Similarly, both the project manager and the PI will blueprint a 15-minute content plan for the introduction/orientation. The introduction/orientation will be conducted virtually by the research team members and the project manager assigned to the implementation. Topics covered include: (1) introduction to CDSS; (2) user workflow; (3) orientation of the CDSS input screen; (4) orientation of the CDSS guidelines screen; and (5) orientation of the CDSS recommendation screen. There will be no evaluation at the end of the introduction/orientation, but time will be dedicated for questions and answers. While no criteria for physician learning are available, it was deemed that the most effective learning for the time-constrained physicians was self-directed through the curated briefing slides, which will be disseminated through email and WhatsApp group.

The physicians will select pre-defined clinical history from the CDSS for imaging recommendations, and they will have the option to either follow or disregard them. We will study the referral practices of the recruited ED physicians in the intervention group over a consecutive period of two months. While ED physicians are not constricted by guidelines, during the study’s second phase, there will be special strategies to improve compliance and to motivate the physicians to adhere to the guidelines. This includes email reminders and the emphasis on the audit process.

The evaluation process in Phase 2 will mimic the pre-implementation evaluation process (Phase 1) to ensure comparable results and data. Essentially, we want to conduct a careful review of the difference between the baseline and post-implementation results. The analysis will include all participating physicians in an intention-to-treat fashion.

#### Outcome 1: Adherence to evidence-based imaging guidelines.

The primary goal is to determine the impact of the CDSS on adherence to evidence-based imaging guidelines in terms of the appropriateness rates of imaging for cervical and lumbar spine X-rays.

For both groups (“Do nothing” and CDSS), we will compare pre-implementation data on the appropriateness of spine imaging referrals with post-implementation data of the same duration (2 months). Multiple readers will evaluate each clinical indication for appropriateness using the local set of diagnostic imaging referral guidelines, with disagreement resolved by consensus.

#### Outcome 2: Clinical effectiveness.

Addressing clinical effectiveness is critical to answering the question, “What is the impact of CDSS on patient outcome(s)?”. To assess the clinical effectiveness of CDSS in the real world, we will examine the clinically significant positive rates of imaging procedures performed, in tandem with the subsequent patient dispositions (i.e., treated and discharge, admission, referral to specialist, referral to physiotherapy and “At Own Risk”) at the end of the individual patient journey in the ED. The radiological reports of associated imaging procedures will be evaluated for significant positive findings that will impact therapeutic decisions, such as fractures in both the cervical and lumbar spines and soft tissue swelling in the cervical spine (i.e., fracture or prevertebral soft tissue swelling – sign of cervical spine jury, were considered positive; all else considered negative). To facilitate comparison in the proportions of positive findings among the categories, we will assess the imaging positivity rates in both the appropriate and inappropriate imaging cohorts. By using a longitudinal approach to monitor patients until their disposition, we will be able to clarify the risks physicians face regarding patient safety and quality of care, particularly in relation to the question: “What impact will following or not following imaging recommendations from the guidelines have on patient outcomes?” [41].

#### Outcome 3: Cost avoidance.

A cost-only analysis will be performed. We will use the associated charges from a local publication on using a bottom-up micro costing approach to calculate the costs of X-rays for spine imaging, specifically the Cervical Spine X-ray at €68.22 and the Lumbar Spine X-ray at €79.85, to estimate cost avoidance [7]. From the dataset, we will estimate the CDSS potential cost avoidance using the formula: “Number of inappropriate imaging X cost of specific imaging”. Additionally, we will also illustrate the potential cost avoidance from the inappropriate imaging.

#### Outcome 4: Radiation dose.

The established literature “Radiology: Patient Exposure from Radiologic and Nuclear Medicine Procedures in the United States: Procedure Volume and Effective Dose for the Period 2006–2016” will serve as the basis for estimating the radiation dose to patients [42]. The publication, based on the International Commission on Radiological Protection (ICRP) Publication 60, indicates an effective dose per scan of 0.36 mSv for X-rays of the Cervical Spine and 1.4 mSv for the Lumbar Spine. We will establish the cumulative effective dose associated with the imaging tests and highlight the comparison of the relative contributions of appropriate and inappropriate imaging with the total number of examinations and the total collective effective dose. We will also evaluate the number of imaging tests avoided (i.e., physician after consulting CDSS followed CDSS recommendations on not referring patient(s) for imaging) and the associated radiation dose prevented using the formula: “effective dose per scan X number of imaging avoided”.

#### Outcome 5: Sustainability.

For this outcome, we will evaluate the impact of CDSS on environmental sustainability. Energy consumption and carbon emissions were approximated from established radiology literature (X-ray Cervical and Lumbar Spine – 0.91 kWh, 1.6 kg CO_2_e) [43–45].

Similar to the thread on radiation dose, we will compare the pre-implementation data on the volume of spine imaging referrals with post-implementation data of the same duration, focusing on the changes in the volume of the overall imaging utilisation before and after the interventions. We will estimate the carbon emissions and energy consumption reduction based on before vs. after implementation data. Similarly, we will also calculate the carbon emissions and energy consumption from imaging tests prevented, using the formula “carbon emissions/energy consumption X number of imaging avoided”.

### Data management plan

No coded/anonymous research data will be sent outside of the study’s institution. We will code the collected data to prevent participant identification. We will store the data in the encrypted/password-protected healthcare cloud (H-cloud), which is the private cloud of Singapore public healthcare. Once the study concludes, we will retain the data for a maximum of seven years before erasing the soft copy. The research data will only be accessible to the PI, who will treat all personal information and results with complete confidentiality. Only authorised personnel will review the data to ensure correct study execution. The PI will also perform data and safety monitoring. The PI will review the recruited data on a monthly basis, and since this study poses only minimal risk, data integrity monitoring is not required. Similarly, this research study does not have any stopping criteria based on efficacy, futility, or safety considerations.

### Safety considerations

There is no expected physical, psychological, social, or economic harms associated with this study. The participants will receive complete information regarding the study design and the scientific rationale underlying the proposed study.

The research team is multidisciplinary and comprises radiographers, EM physicians, and radiologists with sufficient expertise and experience to conduct the research. Data are collected from routine (cervical and lumbar spine X-rays) procedures to avoid unnecessary risk to patients. The PI, who is neither from the ED nor has any relationship with the participants, will perform the data analysis, minimising privacy and confidentiality risks. Additionally, the information will not be used for any purpose except for this research, and that participants will never be identified personally; only group data will be reported.

### Data and statistical analysis

We will describe participant and patient demographics, clinical and imaging characteristics, and study outcomes using standard methods, such as frequencies and percentages for categorical variables and mean, standard deviation, median, and range for continuous variables.

We will use a t-test to examine the outcomes before and after the intervention (“Do nothing” and CDSS). The primary outcome (appropriateness) is binary at the patient level but analysed at the physician (cluster) level. Each physician’s mean appropriateness in the pre- and post-phases will be compared using a paired t-test on within-physician differences; if assumptions of normality are materially violated, analyses will be repeated with a variance-stabilising transformation.

To complement this, patient-level multilevel logistic regression models will be performed with a random intercept for physician and fixed effects for phase, intervention arm, and their interaction. Patient-level (age, sex, indication type) and physician-level (specialty) covariates will be included to adjust for potential confounding. Both unadjusted cluster-level and adjusted multilevel estimates will be reported side by side. Where possible, we will present all effects with confidence intervals.

### Status and timeline

Initial data collection will start on 01 May 2025. We aim to complete data collection by 30 November 2025 and perform all analysis by 31 December 2025.

## Discussion

This study protocol outlines the methodology for a pragmatic controlled before-after study involving a single centre in Singapore. The study evaluates the impact of implementing a CDSS on the appropriateness of X-ray examinations of the cervical and lumbar spines in the ED, in contrast to the current practice without such a system. The impact on associated outcomes will also be elucidated.

The EBI of a CDSS provides ED physicians with evidence-based information and actionable decision support for imaging decisions, addressing gaps in the availability of imaging referral guidelines and the lack of guideline use. This is a critical step toward ensuring the appropriate use of radiation, avoiding unnecessary exposure, and achieving imaging efficiency in a safe and high-quality manner.

However, a recent randomised clinical trial which investigated the impact of a CDSS on the appropriateness of medical imaging ordering by physicians concluded that the CDSS did not reduce the number of inappropriate imaging requests [39]. We believe this conclusion is misleading and does not accurately reflect the potential of CDSSs to improve imaging appropriateness. There were suggestions that the lack of changes lies not with the concept of CDSSs or guidelines but rather with the specific implementation, lack of context analysis, and usability of the system in that study. Indeed, it underscores the need for transdisciplinary effort to develop and implement CDSSs into clinical workflows, provide clear and concise recommendations, and address clinician concerns about autonomy. This study has the potential to shed light on these issues.

Unsurprisingly, despite successful implementation at one location or the existence of consensus evidence, practice change can be impeded by multiple factors, including competing policy priorities, entrenched cultural norms, and mismatched resources, among others [46]. Recognising the importance of implementation, we adhered to implementation science process models to guide our implementation of the standalone CDSS. Firstly, we established baseline data on the prevalence of low-value cervical and lumbar spine imaging [22,47]. Next, we conducted various mixed-methods and qualitative studies with the ED physicians to assess their readiness for practice changes and to develop implementation strategies that will encourage uptake of CDSS [41,48]. Simultaneously, we also sought to address their concerns of clinical effectiveness and/or risks of using imaging referral guidelines by elucidating that these guidelines were effective in excluding positive findings in traumatic and non-traumatic patients [38]. Additionally, we ensured that we have champions that help facilitate and promote practice changes while constantly engaging institution leadership for support and guidance. Collectively, these are geared for a more successful implementation.

Utilising JBI’s seven-phase process model enables us to systematically design and develop our implementation in distinct phases [47]. While there are numerous theories, models, and frameworks (TMFs) in implementation science to enhance the implementation process, the seven-phase process model was selected for its emphasis on collaborative partnerships and contextual comprehension at each phase of the implementation cycle, a principle we also firmly had conviction in.

Although there is a variety of evidence from America and Europe, literature addressing the deployment of CDSS in the Asia region is scarce. This is not surprising, given that clinical environments across Asia neither mandate nor extensively implement CDSS. Moreover, this study presents difficulties due to the rotation of ED physicians, especially junior ones, through multiple healthcare institutions during their 60-month residency, resulting in inconsistent engagement with CDSS. Consequently, this study is likely to be of low priority and challenging to conduct. Therefore, by engaging various change agents and leaders and adopting the most practical approaches, we are quietly confident that we will be able to contribute to this body of knowledge.

### Strengths

One of the strengths of this study lies in our approach to implementation, in which we will apply implementation science principles to guide the EBI’s deployment. We will utilise JBI’s 7-phase process model to facilitate and direct the integration of evidence into practice, specifically the integration of CDSS into the practice of ED physicians [47]. Notably, we have identified problems with stakeholders, engaged change agents and assessed the context and readiness of ED physicians for change [48]. Through these phases, we have identified barriers and opportunities in the ED/healthcare systems as vital for reducing low-value imaging, developed a better understanding of how physicians make sense of the CDSS intervention, engaged key leadership for endorsement of technological interventions, and ensured shared agreement and engagement where ED physicians, radiologists, and radiographers are involved in the implementation project: developing guidelines, modifying CDSS, user acceptance testing, and developing implementation strategies.

Additionally, we will use a controlled before-after study design to measure changes in the outcomes linked to the intervention. By having a control group, it provides more confidence in the study’s results through the elucidation of changes over time (i.e., attributing causal inference to the intervention).

Although CDSS are designed to improve quality of care and reduce costs, a review has shown no change in the total volume of advanced imaging orders and small changes in appropriateness [49]. This can be a result of suboptimal implementation, which negatively impacts the uptake of evidence in real-world practice [50]. Literature indicates that demonstrating the effectiveness of an EBI does not ensure its integration into routine practice, as uptake is significantly influenced by contextual factors (e.g., environment, specific setting, individual characteristics) [37]. This study will build on previous findings and will provide comprehensive data on the impact of this EBI in real-world setting on healthcare systems and patients while accounting for barriers and facilitators in the implementation process. Drawing from unpublished results from a locally conducted study that assessed the context and readiness of ED physicians to promote evidence-based imaging referral guidelines, targeted implementation strategies will be in place to bridge the evidence-to-practice gap.

We have intentionally adopted a pragmatic methodology for this study to gather empirical insights regarding the use and effects of CDSS in healthcare. In our study, our recruitment approach provides the best opportunity for rapid recruitment of ED physicians during the transition from another institution to the study’s ED, which occurs during the study period. This study will be informative regardless of the outcome.

### Limitations

The study design is however also subject to several limitations, which are minimised where possible.

An often-discussed potential drawback of controlled before-after studies is methodological weakness, which results in a higher risk of bias, as there may be unidentified differences between the intervention and control groups that may affect changes in the outcome measure. For instance, the performance at baseline for both groups may differ, and subsequent analyses may be inappropriate, as the baseline imbalance suggests that the control group is not comparable [50]. This could render any apparent effect of the intervention spurious. Indeed, it is often difficult to identify a comparable control group [50]. However, we will endeavour to minimise these differences by ensuring that the participating ED physicians shared similar clinical experiences when recruited, i.e., previous ED postings prior to this study. Nonetheless, it is prudent to acknowledge that there is no direct comparison between the control and intervention groups, therefore the estimate of effect attributable to the intervention should be interpreted with caution. However, we will ensure that a table of baseline comparisons is available for readers, which will facilitate clinical judgement in determining whether any of the differences are substantial enough to have influenced the outcome [51].

Furthermore, in this study, the EBI may be influenced by the Hawthorne effect, which could result in an overestimation of the intervention’s efficacy [52]. However, we contend that the impact will be negligible, as prior quality assurance audits on X-ray lumbar spine, conducted after the implementation of institutional imaging guidelines, revealed minimal variations in both volume and appropriateness, even with ED physicians informed of the ongoing audits.

A possible constraint in the efficacy of CDSS is the emergence of alert fatigue among physicians, leading them to disregard feedback from the CDSS or perceive it as an onerous regulation rather than a tool to enhance the appropriateness of imaging referrals [53]. Moreover, to bypass the standalone CDSS, ED physicians may attempt to “game the system” by selecting indications that will result in their imaging referral being deemed *Appropriate.* We acknowledge these constraints and therefore seek to adhere to the “Ten Commandments for Effective Clinical Decision Support” to maximise the success of the implementation [54].

Simultaneously, effective doses are estimates and may vary depending on local equipment, protocols, patient body habitus, and radiographer practices. Therefore, the estimates for the cumulative effective doses may differ from other authors. Similarly, the estimates for energy consumption and carbon emissions may vary due to hospital equipment efficiency, energy sources, and operational practices. However, with the complexity involved in estimating these values, we believe the impact will be minimal in terms of magnitude, as we adopted estimates from published works in journals such as Radiology and The Lancet, while also including estimates from an international organisation – ICRP [55].

Our study may also be susceptible to inherent bias. This encompasses biases such as selection and history. To minimise selection bias, the research team will recruit newly posted junior ED physicians whose residency matches are decided by a neutral party (MOH Holdings Pte Ltd., Singapore). Similarly, to mitigate history threat, all changes that could impact the outcomes would be accounted for, and the time span between the before and after is minimised to reduce the effects of trends, which in our case is each two months in close succession [56].

Additionally, the relatively small sample size and single-centredness will limit the external validity of this study. Using implementation science principles in our study, we are able to create a “culturally adapted” intervention that takes into account feedback from ED physicians. This resulted in a refined and more acceptable CDSS for end users, particularly in Singapore. This could serve as a blueprint for other countries to adopt and adapt our approach in implementation of CDSS.

Lastly, it has been recognised that the impact of standalone CDSS is often lower than that of integrated systems due to the extra work required by users to obtain an imaging recommendation amid a busy clinical environment. However, there is a limitation surrounding the local information technology infrastructure, whereby a change in the electronic medical record system is expected shortly, rendering integration work unfeasible.

Despite the limitations, the study is maximising the opportunity to draw meaningful conclusions that will aid future studies and our own research in the implementation of CDSS. Specifically, this study will serve as baseline data and evidence for implementing and adopting integrative CDSS within the local health system/healthcare cluster. Plans are currently in progress for an exploratory study on a pilot integrative CDSS to be implemented at the point of care in academic hospitals and primary care settings, addressing the challenges associated with standalone CDSS, such as workflow burdens and “alert fatigue”.

## Conclusion

The findings from this study will provide insights into the CDSS’s impact and effect in the ED setting. It contributes to the body of knowledge on the effectiveness of CDSS, particularly in the ED setting, which may be generalisable to other countries. Additionally, it generates local evidence that facilitates a review of current practice and, consequently, leads to practice change. Despite primarily focusing on the ED setting, the findings hold potential for wider implementation or adoption, which includes primary care settings.
